# Supplementary material for: Chip-scale reconfigurable carbon nanotube physical unclonable functions
Source: Nat Commun. 2025 Sep 30;16:8705. doi: 10.1038/s41467-025-63739-x (PMC12484790; doi:10.1038/s41467-025-63739-x)
Supplement: Supplementary file 1 — Supplementary Information [file 41467_2025_63739_MOESM1_ESM.pdf]

Supplementary Information for

Chip-scale reconfigurable carbon nanotube physical unclonable functions

Yang Liu[1,2,†], Jingfang Pei[1,†], Yingyi Wen[1], Lekai Song[1], Songwei Liu[1], Pengyu Liu[1], Wenyu Cui[3], Zihan Liang[4], Teng Ma[3], Xiaolong Chen[4], Guohua Hu[1,*]

[1]Department of Electronic Engineering, The Chinese University of Hong Kong, Shatin, New Territories, Hong Kong S. A. R., China
[2]Shun Hing Institute of Advanced Engineering, The Chinese University of Hong Kong, Shatin, New Territories, Hong Kong S. A. R., China
[3]Department of Applied Physics, Hong Kong Polytechnic University, Hung Hom, Kowloon, Hong Kong S. A. R., China
[4]Department of Electrical and Electronic Engineering, Southern University of Science and Technology, Xueyuan Avenue, Shenzhen 518055, China

†Equal contribution to this work
*Correspondence to: Guohua Hu (ghhu@ee.cuhk.edu.hk)

This file contains:

Supplementary Note 1
Supplementary Figures 1-29
Supplementary Tables 1-3
Supplementary References

Supplementary Note 1

In our work, having proved the capability of our PUFs to enhance hardware security, we explore their potential application in self-driving. This exploration is conducted on OMNeT++ platform. OMNeT++ is a widely adopted open-source platform for building network simulators to implement and study real-time road traffic and vehicle communication.[1]

As schematically illustrated in Supplementary Fig. 26, when building the vehicular network on OMNeT++, each vehicle is equipped with a carbon nanotube PUF chip, and the secured communication among the vehicles can be phased into *Reconfiguration*, *Authentication*, and *Communication* stages.

- *Reconfiguration* (Supplementary Fig. 26a): A vehicle equipped with a PUF chip initiates a request to the trust authority to update its identity. The trust authority checks and verifies the current identity of the vehicle and sends a challenge to the vehicle. Upon receiving the challenge, the PUF equipped on the vehicle generates a new challenge-response pair to create a fresh, unclonable identity which is then verified and stored by the trust authority. This *Reconfiguration* process is designed to ensure safe and dynamic identify update of the vehicles in the vehicular network. The *Reconfiguration* implementation protocol is detailed in Supplementary Fig. 27.

- *Authentication* (Supplementary Fig. 26b): The trust authority issues a challenge to the PUF equipped on the vehicle, and the PUF upon receiving the challenge generates and returns its response to the trust authority. As such, the vehicle allows the trust authority to verify its identity by checking the response sent back. This completes a mutual-verification handshake in less than 1 ms, an order of magnitude faster than the conventional approaches.[2] Due to the unique, unclonable nature of the PUFs, attacking becomes virtually impossible, enabling secure access control. The *Authentication* implementation protocol is detailed in Supplementary Fig. 28.

- *Communication* (Supplementary Fig. 26c): Using the communication between Vehicle A and B as an example, Vehicle A sends message with its PUF-derived session token and a timestamp to Vehicle B, and Vehicle B then forwards the packet to the trust authority for a sub-50-μs timestamp verification. Upon receiving the verification, Vehicle B generates and sends message with its PUF-derived session token and a timestamp to Vehicle A. Again, Vehicle A then forwards the packet to the trust authority for verification. Upon receiving the verification, Vehicle A completes a mutual-verification handshake with Vehicle B before their communication. As such, vehicles use PUFs to establish secure communication with timestamps and secure cryptographic tokens verified by the trust authority. This ensures the hardware integrity and prevents replay attacks. The *Communication* implementation protocol is detailed in Supplementary Fig. 29.

In this modelling, PUFs are embedded in the vehicular network by embedding a *PUF Module* in each vehicle module. Specifically, the *PUF module* is allowed to feature the characteristics of our carbon

nanotube PUFs in generating 108-bit responses upon challenges, including the reconfigurability and input-output response times.

To specify, during the modelling, *Simulation of Urban MObility* (SUMO) simulates the movement of a self-driving vehicle. When the vehicle enters a set range, wireless vehicle communication can be activated, and the *PUF module* begins to work. For *Communication*, the trust authority sends a challenge to the vehicle, and the *PUF module* in the vehicle outputs a 108-bit response. Since the *PUF module* simulates the response behavior of our carbon nanotube PUFs to the challenge, there is a time delay of about 1 ms in generating the 108-bit response. Time delay is a key parameter in the *Communication* process, which can affect the freshness of the message and the success rate of attacking. According to the above discussion and results we show in Fig. 6, the median end-to-end time delay measured by the time-accurate event-driven simulator is about 12 ms (~100 ms in the worst case for up to 100 vehicles). Attacking the response in such a short time is nearly impossible (Fig. 5e), so it can ensure the security of message transmission. In addition, the 108-bit response is embedded in the message transportation in the *Vehicular ad hoc network* (VANET) after passing through the Elliptic Curve Cryptography (ECC) based encryption. Based on the *Communication* implementation protocol (Supplementary Fig. 29), the ECC-based encryption cost (around 0.87 ms per vehicle) is very low, so it can enable lightweight communication.

The complete secured communication between the vehicles, including the *Reconfiguration*, *Authentication*, and *Communication* stages, are modelled and presented in Supplementary Movie 1. The result demonstrates that our PUFs can be embedded into the existing protocol flows and deliver hardware security with substantially reduced communication and computational overhead as well as communication delays (Fig. 6). For example, we approach achieves a typical time delay of ~12 ms for networks of 10 to 100 vehicles, with a maximum delay of ~100 ms, well within the 50 ms self-driving requirement, and enables lightweight authentication and communication with only 324 bits per vehicle for communication and 1,544 bits for authentication, alongside a low computational cost of ~0.87 ms per vehicle.

Supplementary Figures

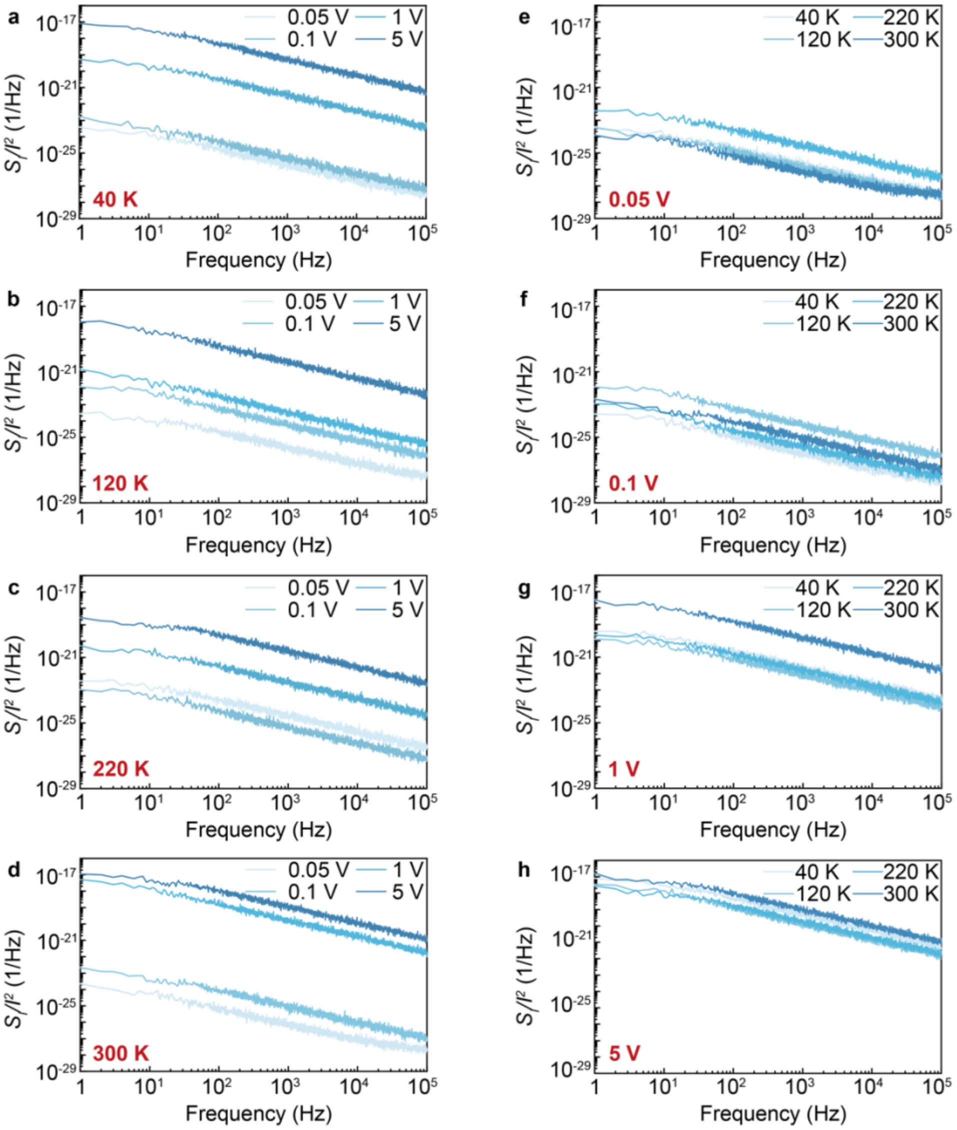

Supplementary Fig. 1. Noise power density spectra of the carbon nanotube transistors. (a-d) Noise power density spectra as measured and presented at 40 K, 120 K, 220 K, and 300 K, proving 1/*f* noise and that the 1/*f* noise is stable and invariant against the temperature variation; (e-h) Noise power density spectra as measured and presented with 0.05 V, 0.1 V, 1 V, and 5 V bias, proving 1/*f* noise and that the 1/*f* noise levels increase with the bias. The 1/*f* noise with the temperature-independent and bias-dependent characteristics suggests the noise arises from charge trapping dynamics in carbon nanotubes and that the charge trapping dynamics are robust in low and even cryogenic temperatures.

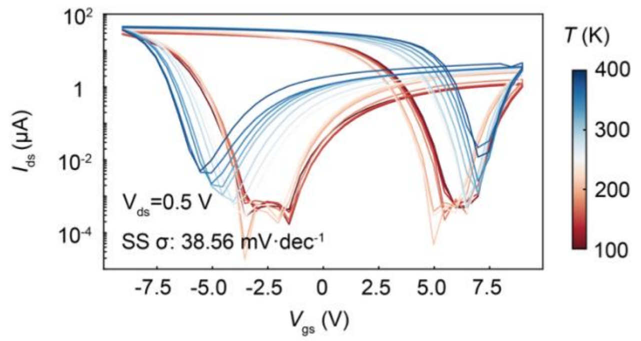

Supplementary Fig. 2. Transfer curves of the carbon nanotube transistors. The standard deviation of the subthreshold swing as extracted from the transfer curves is 38.56 $mV \cdot dec^{-1}$. The transistors are measured from 100 K to 400 K, with a 20 K interval.

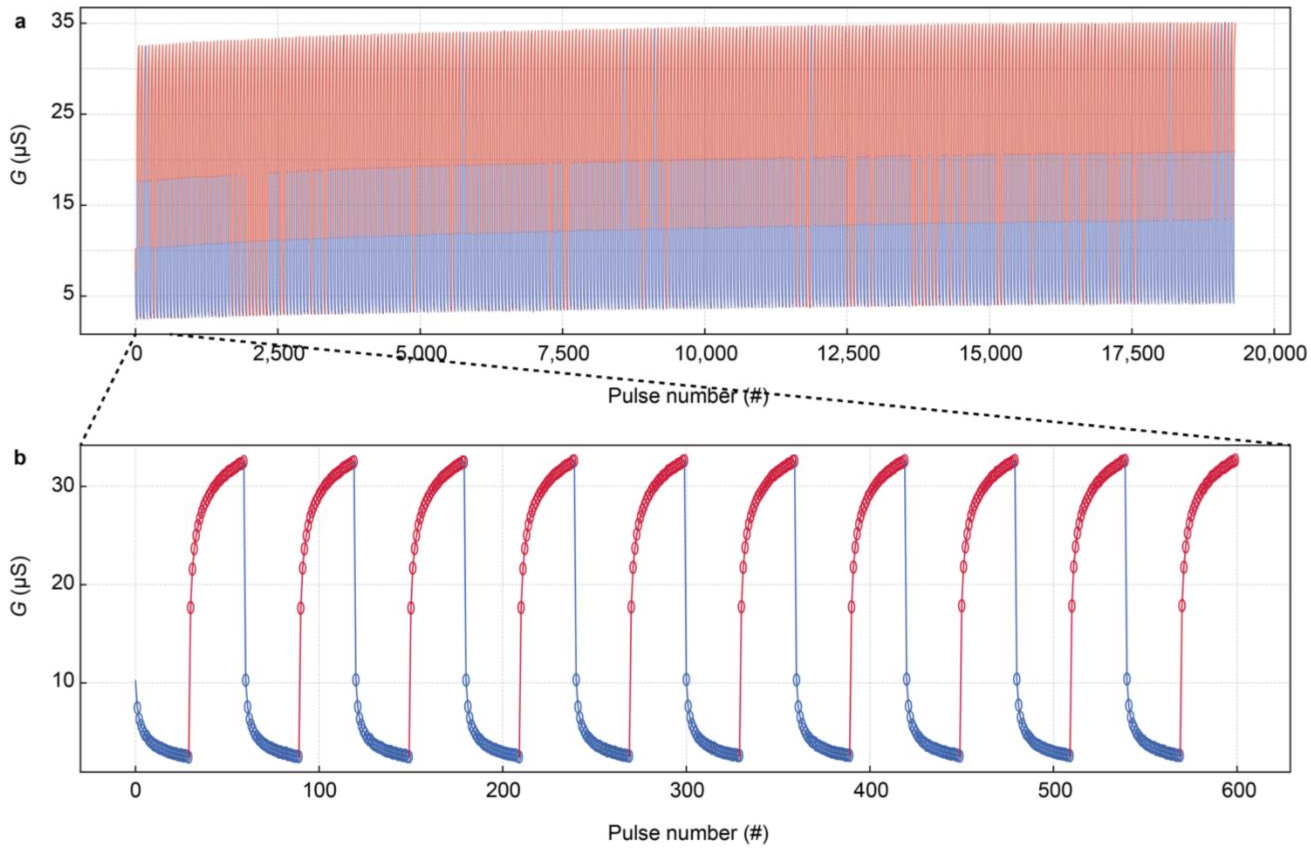

Supplementary Fig. 3. Cycle-to-cycle operational reproducibility of the carbon nanotube transistors. (a) The conductance profile probed from a typical transistor when modulated with alternating positive-negative voltage pulses V_{gs}. 30 consecutive positive pulses V_{gs} (amplitude 3 V, duration 10 µs) are applied to reset the transistor, and 30 consecutive negative pulses V_{gs} (amplitude -3 V, duration 10 µs) are applied to configure the transistor. The red dots correspond to the conductance in configuring, and the blue dots correspond to the conductance in resetting. The cycle-to-cycle variation in conductance modulation is <6.8%. A drain voltage V_{in} of 0.5 V DC is applied to probe the conductance. (b) Zoomed-in conductance profile.

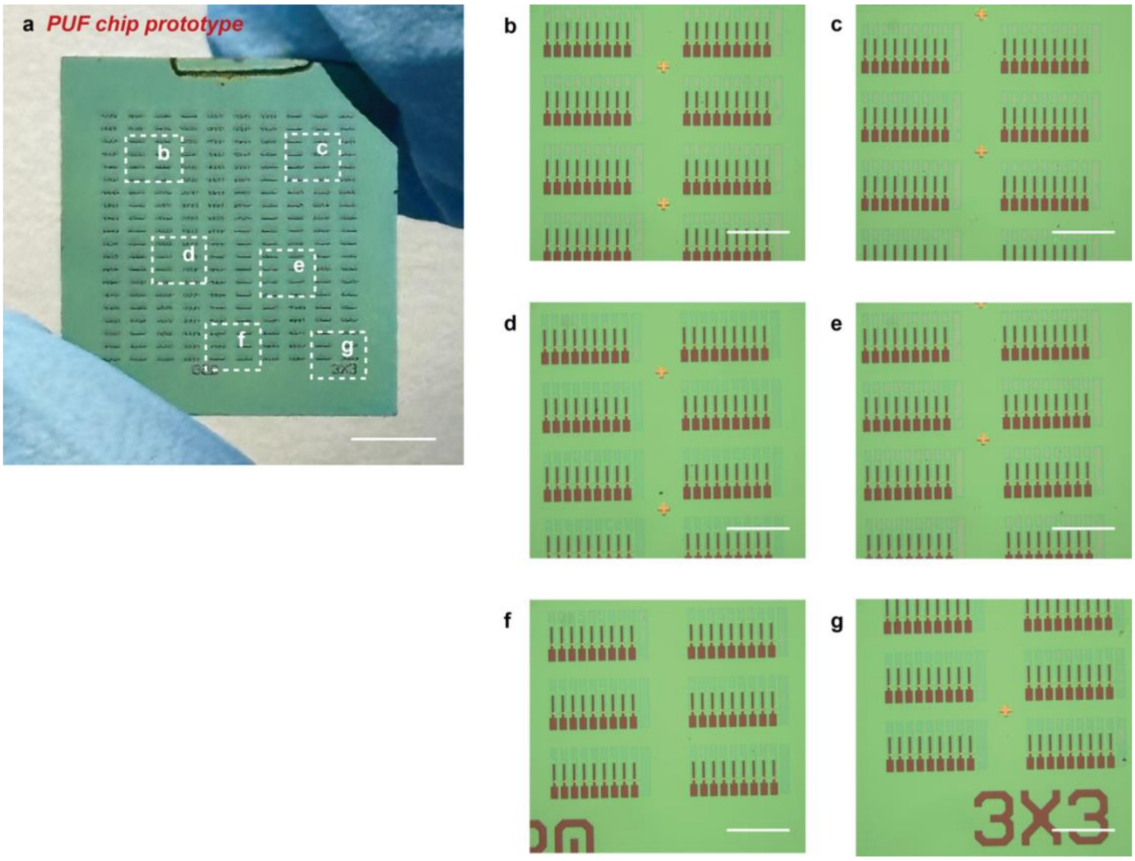

Supplementary Fig. 4. PUF chip prototype. (a) Photo of a PUF chip prototype, with (b-g) optical microscopic images of the PUFs. The PUF chip prototype integrates 40 PUFs. Scale bar – (a) 0.4 cm, (b-g) 500 μm.

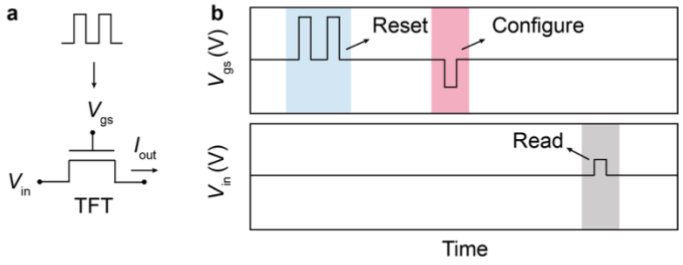

Supplementary Fig. 5. Caron nanotube transistor operation of the PUFs. (a) Diagram and (b) the corresponding pulse profiles showing the operation of the transistors. Voltage pulses V_{gs} are first applied onto the gate of all the individual transistors for resetting and configuration, and voltage pulses V_{in} are then applied onto the common drain of the transistors as the challenge. Current outputs I_{out} are read from the source of the individual transistors as the responses.

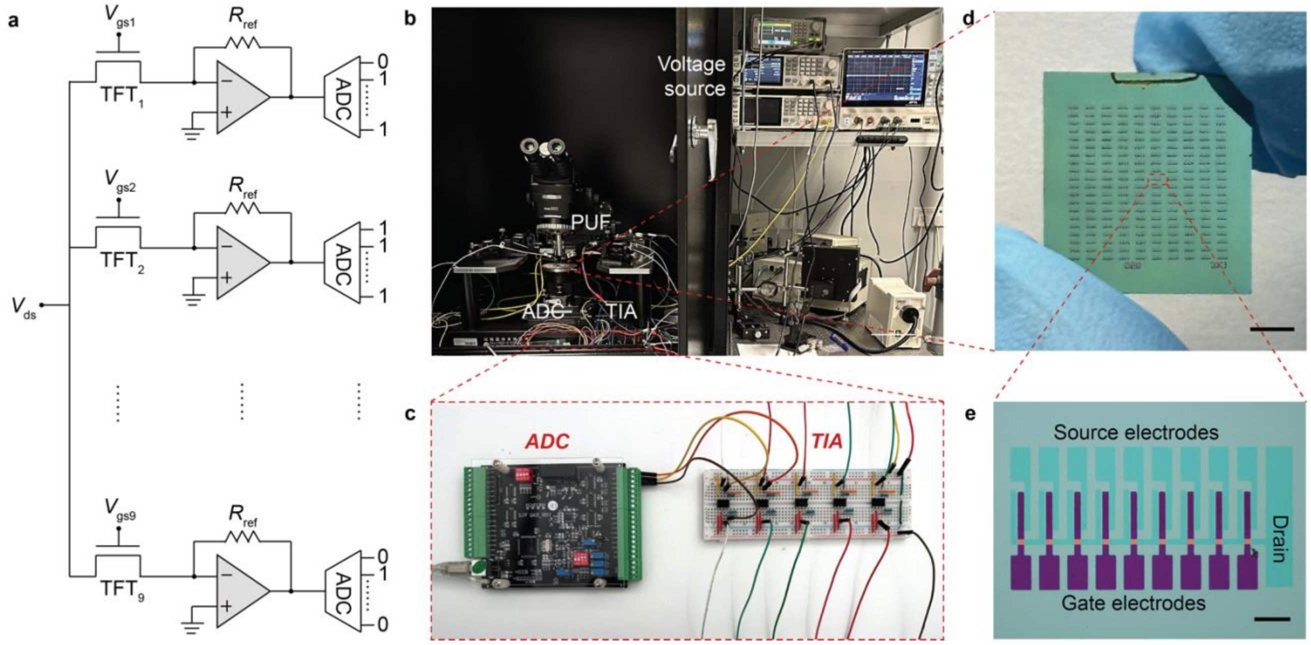

Supplementary Fig. 6. Hardware operation of the PUFs. (a) Schematic operation of the PUFs with an ADC testing board, and (b-e) the corresponding hardware testing setup and operation. Each PUF consists of 9 transistors that are connected via a common drain. The PUFs are mounted on a probe station for the operation, with the gates connected to the pulse measure unit (PMU) of Tektronix Keithley 4200A-SCS for the state modulation, the common drain connected to an arbitrary waveform generator (Siglent SDG7032A) and a digital storage oscilloscope (SDS2354X) for input of pulsed voltage signal as the challenge, and the sources connected to the ADC testing board (Zhengzhou Hengkai Electronic Technology Co.) via trans-impedance amplifiers (TIA) modules for output of pulsed current response and the following conversion and binarisation. The challenge pulse width is configured as 1 μs and 1 MHz, and the amplitudes are configured as requested by the primitive generations. Each response pulse is converted and binarised into 12 binary digits. Upon operation, the states of the individual transistors are first modulated to configure the PUF state; after the configuration, the challenge is applied, and the PUF output response is then binarised and digitised for the primitive generation. Scale bar – (b) 10 cm, (d) 0.3 cm, (e) 100 μm.

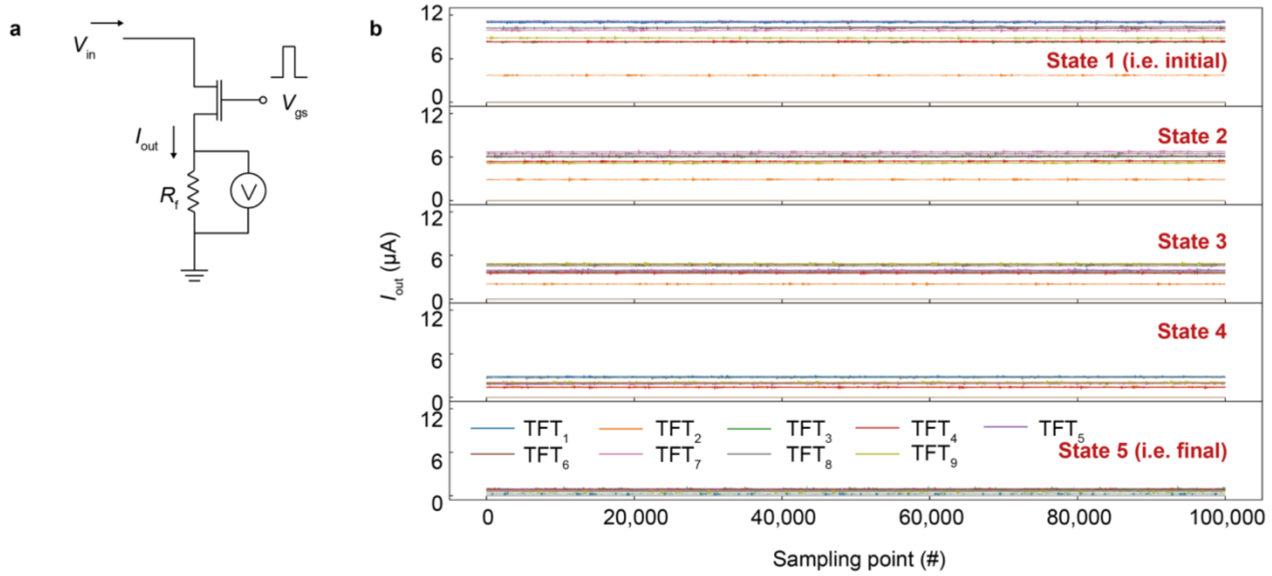

Supplementary Fig. 7. Current outputs from the 9 individual carbon nanotube transistors in a typical PUF through 100,000 challenge times. (a) Circuit diagram showing the test. (b) Current outputs I_{out} from the 9 transistors in the PUF. All the 9 individual transistors are configured at the initial, final, and three other states by gate pulses V_{gs}. V_{gs} is 0 V, -3 V, -5 V, -7 V, and -9 V to configure the State 1-5, respectively. Current outputs I_{out} are probed under the challenge, i.e. drain V_{in} with amplitude 0.5 V DC is composed of 100,000 sampling times. All the 9 transistors in these 5 states give highly stable outputs through the 100,000 challenge times.

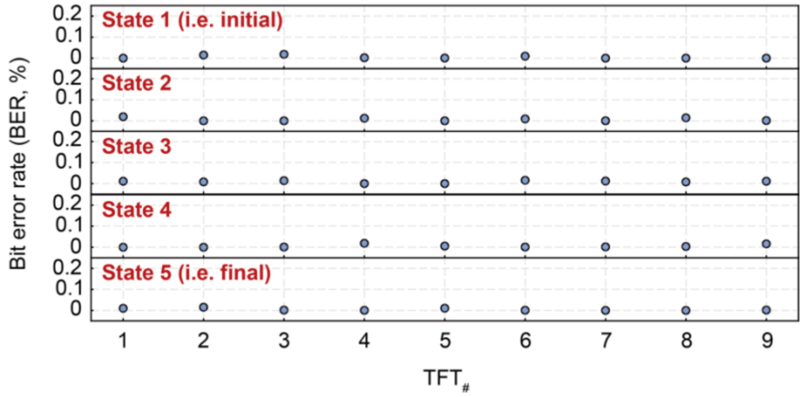

Supplementary Fig. 8. Berror rate (BER) of operating our PUFs. BER of all the 9 individual transistors of a typical PUF in the 5 states. The BER is <2% for all the transistors in all the states.

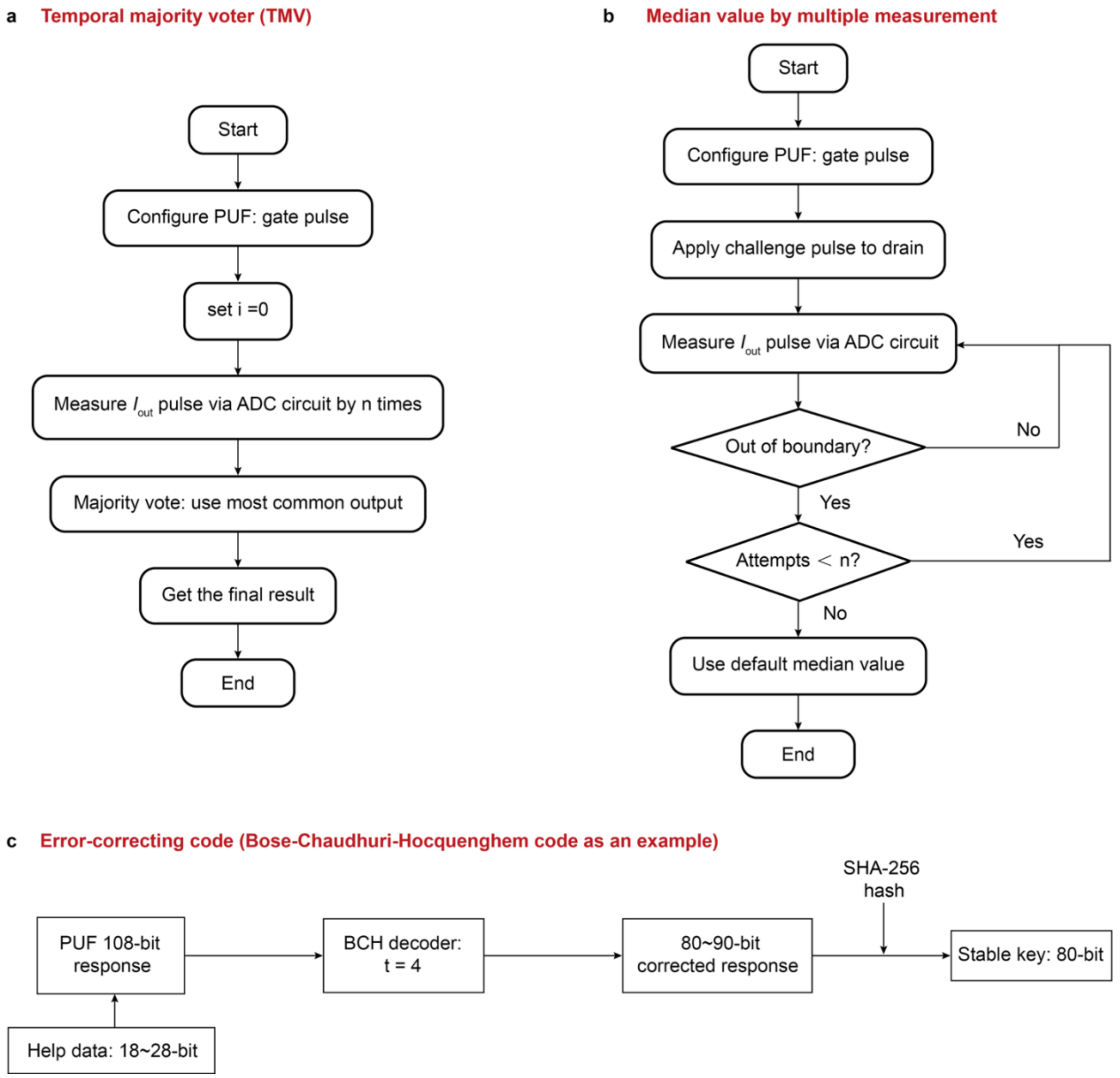

Supplementary Fig. 9. Algorithm improvements for our PUF operation. (a) *Temporal majority voter (TMV)*, repeatedly measuring the response and selecting the most frequent output as the result. (b) *Median value by multiple measurement*, checking if the measured PUF response lies within acceptable bounds and using the median value as the result. (c) *Error-correcting code process*, decoding the PUF response with error bits using helper data to generate a corrected response, which is then hashed (e.g., via SHA-256) to produce a stable cryptographic key.

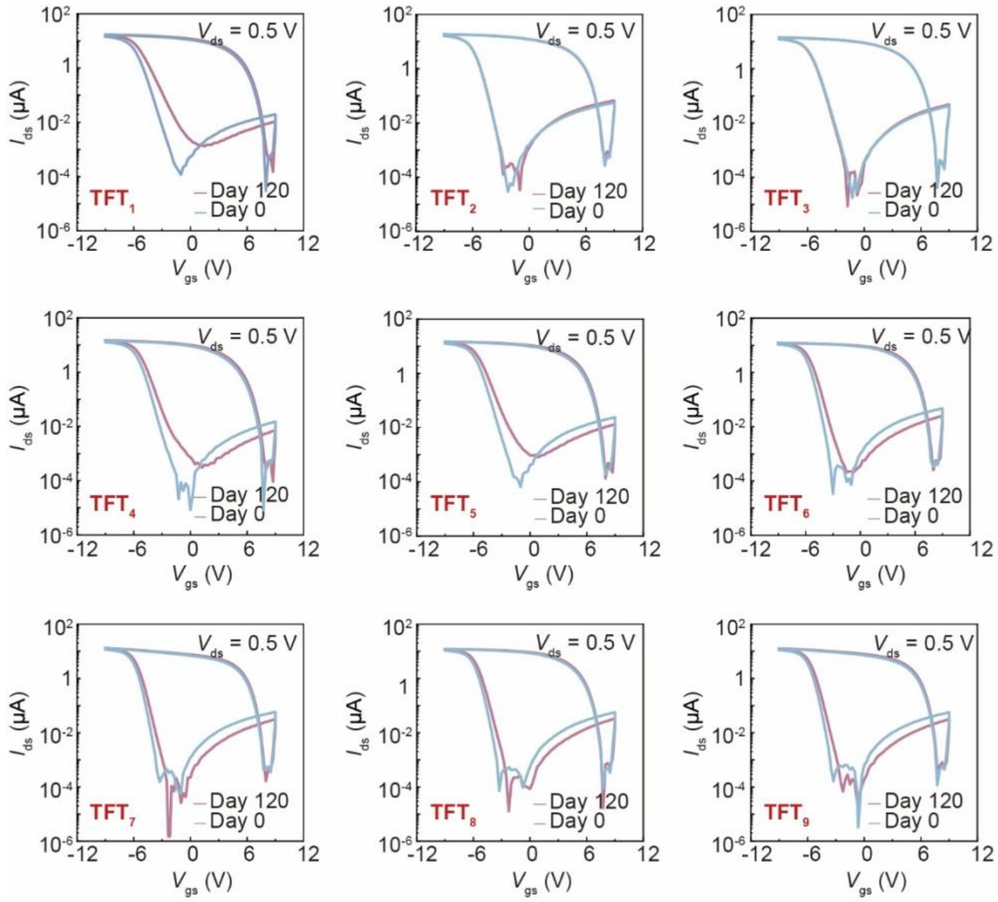

Supplementary Fig. 10. Transfer curves from the individual transistors in a PUF. The PUF is kept at ambient conditions without encapsulation or protection. The tests are conducted on the first day and the 120th day, proving an operational stability of the transistors.

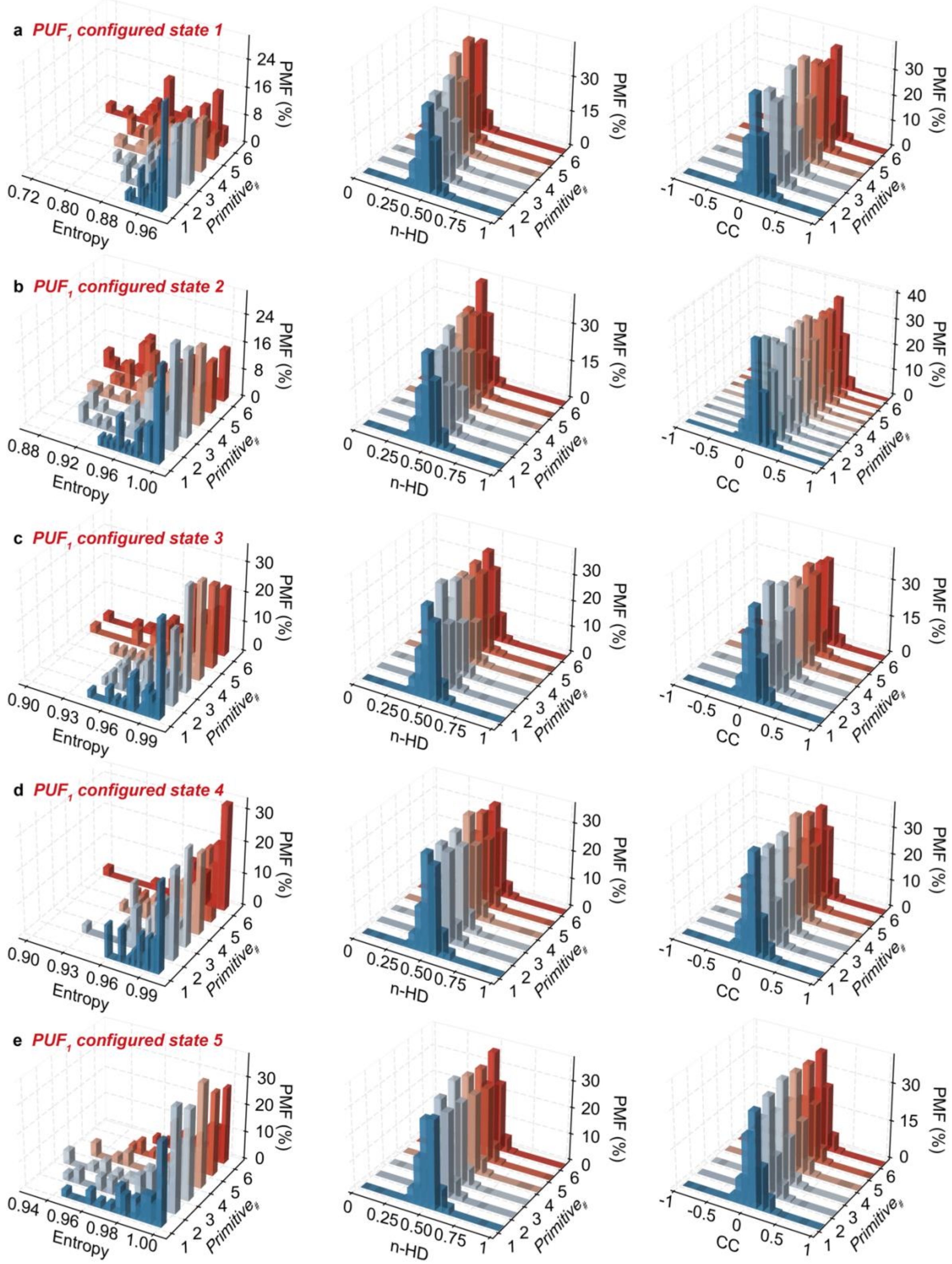

Supplementary Fig. 11. Physical unclonability. Entropy, normalized Hamming Distance (n-HD), and correlation coefficient (CC) of PUF_1 at five randomly configured states. The above results show ideal randomness, uniqueness, and irrelevance from one another and in configuration operations.

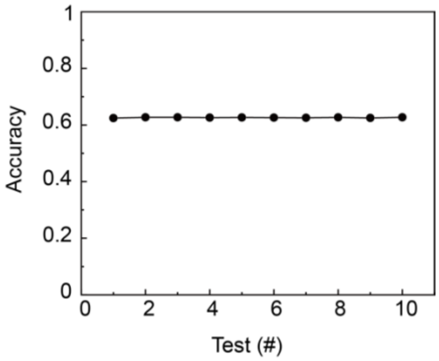

Supplementary Fig. 12. Prediction accuracy of XGBoost attacking in 10 tests. The averaged prediction accuracy for the 10 tests is 62.61%, proving the resilience of the PUFs against XGBoost attacking.

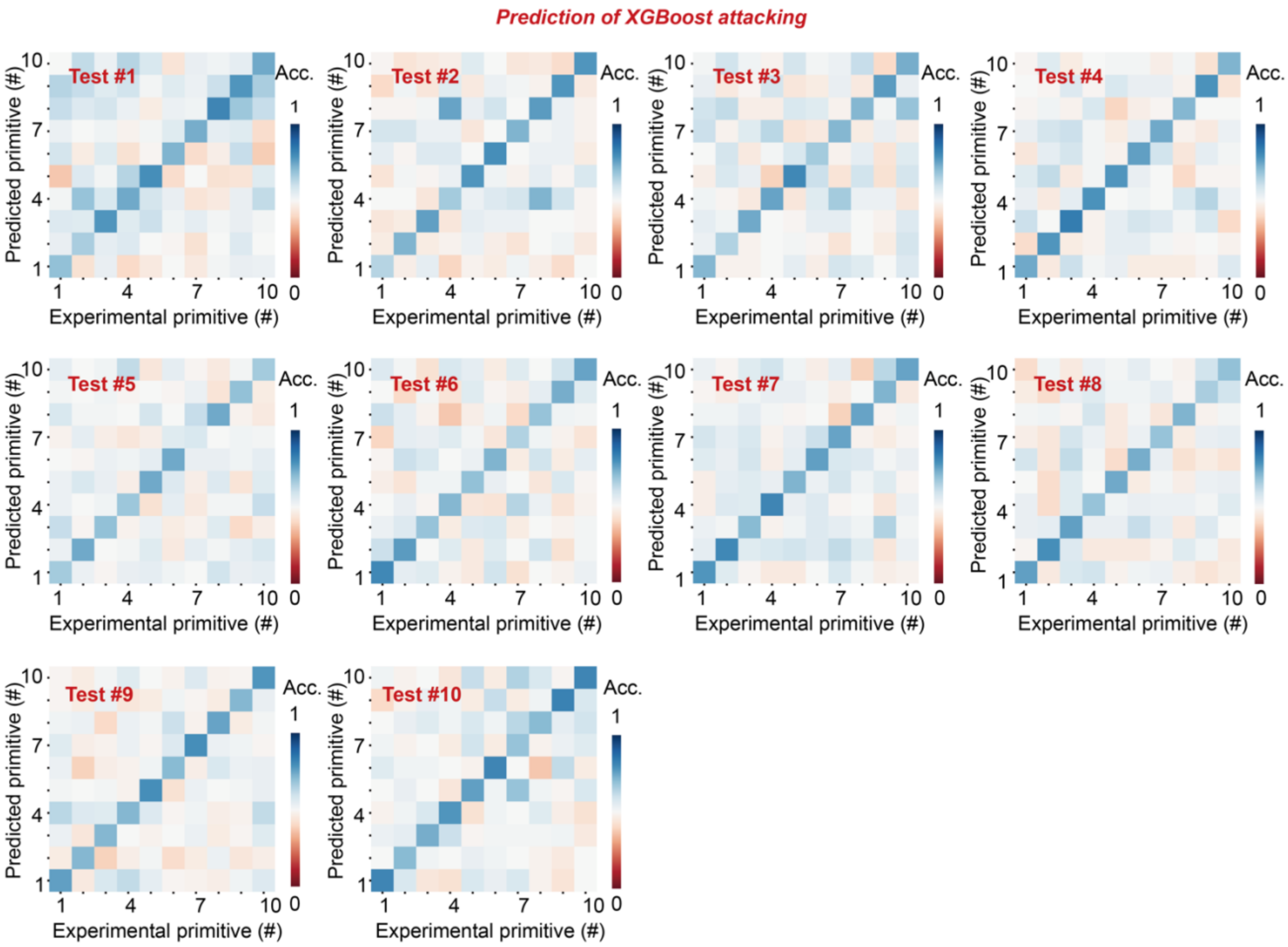

Supplementary Fig. 13. Prediction accuracy of XGBoost attacking in 10 tests. Prediction accuracy (Acc.) matrix of 10 experimental primitives and 10 predicted primitives as randomly and respectively selected from the 10 tests for comparison and evaluation, proving that the predicted primitives fail to catch the characteristics of the experimental primitives, i.e. proving the resilience of the PUFs against XGBoost attacking.

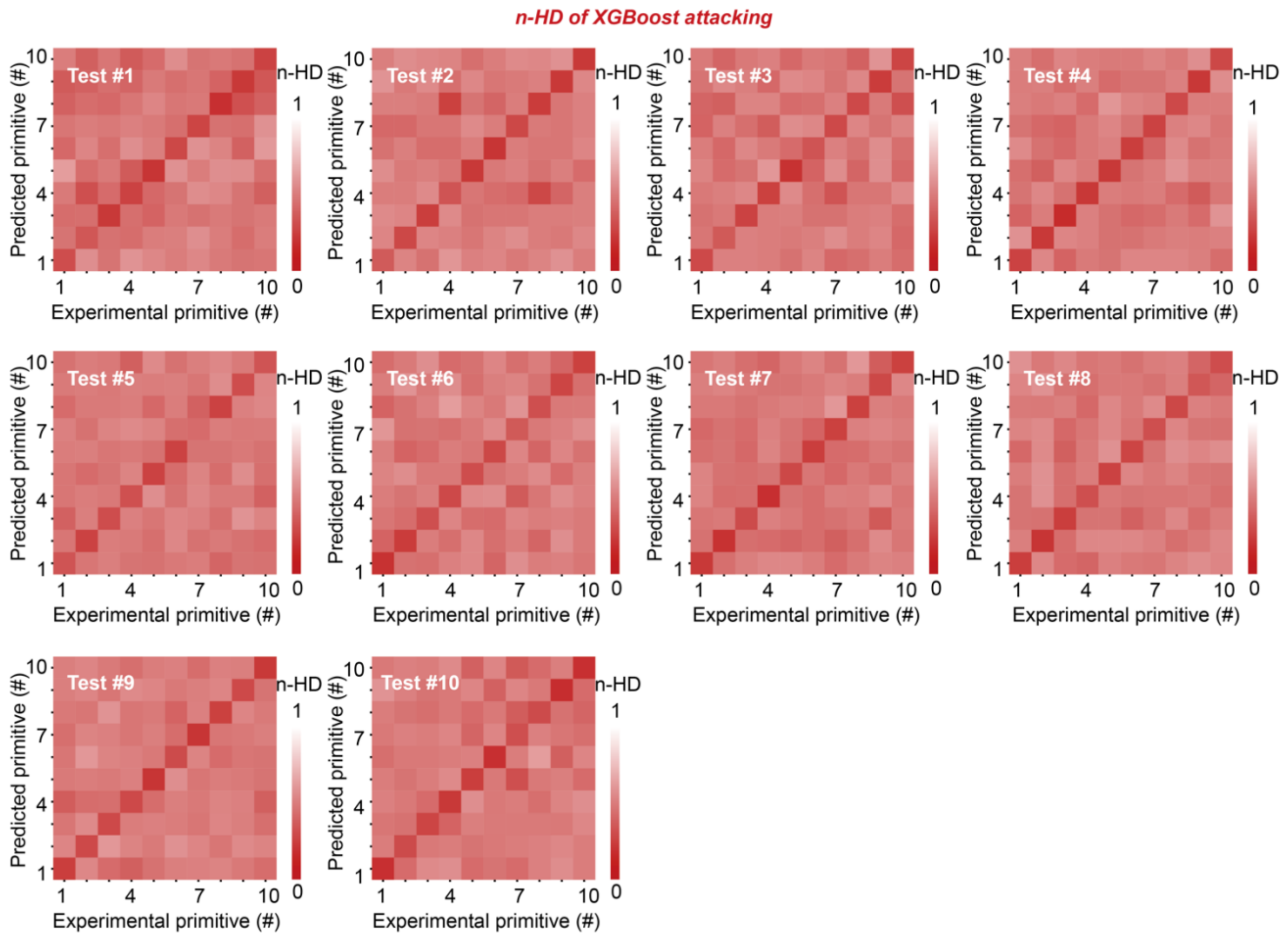

Supplementary Fig. 14. n-HD of XGBoost attacking in 10 tests. n-HD matrix of 10 experimental primitives and 10 predicted primitives as randomly and respectively selected from the 10 tests for comparison and evaluation, proving that the experimental and predicated primitives are highly unique to each other, i.e. proving the resilience of the PUFs against XGBoost attacking.

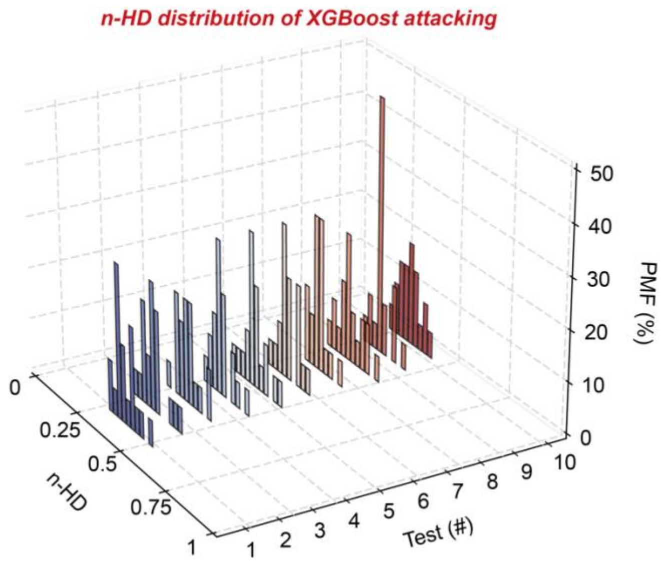

Supplementary Fig. 15. Distribution of n-HD of XGBoost attacking in 10 tests. Distribution of n-HD as analysed from 10 experimental primitives and 10 predicted primitives as randomly and respectively selected from the 10 tests for evaluation. n-HD is all approaching 0.37, proving that the experimental and predicated primitives are highly unique to each other, i.e. proving the resilience of the PUFs against XGBoost attacking.

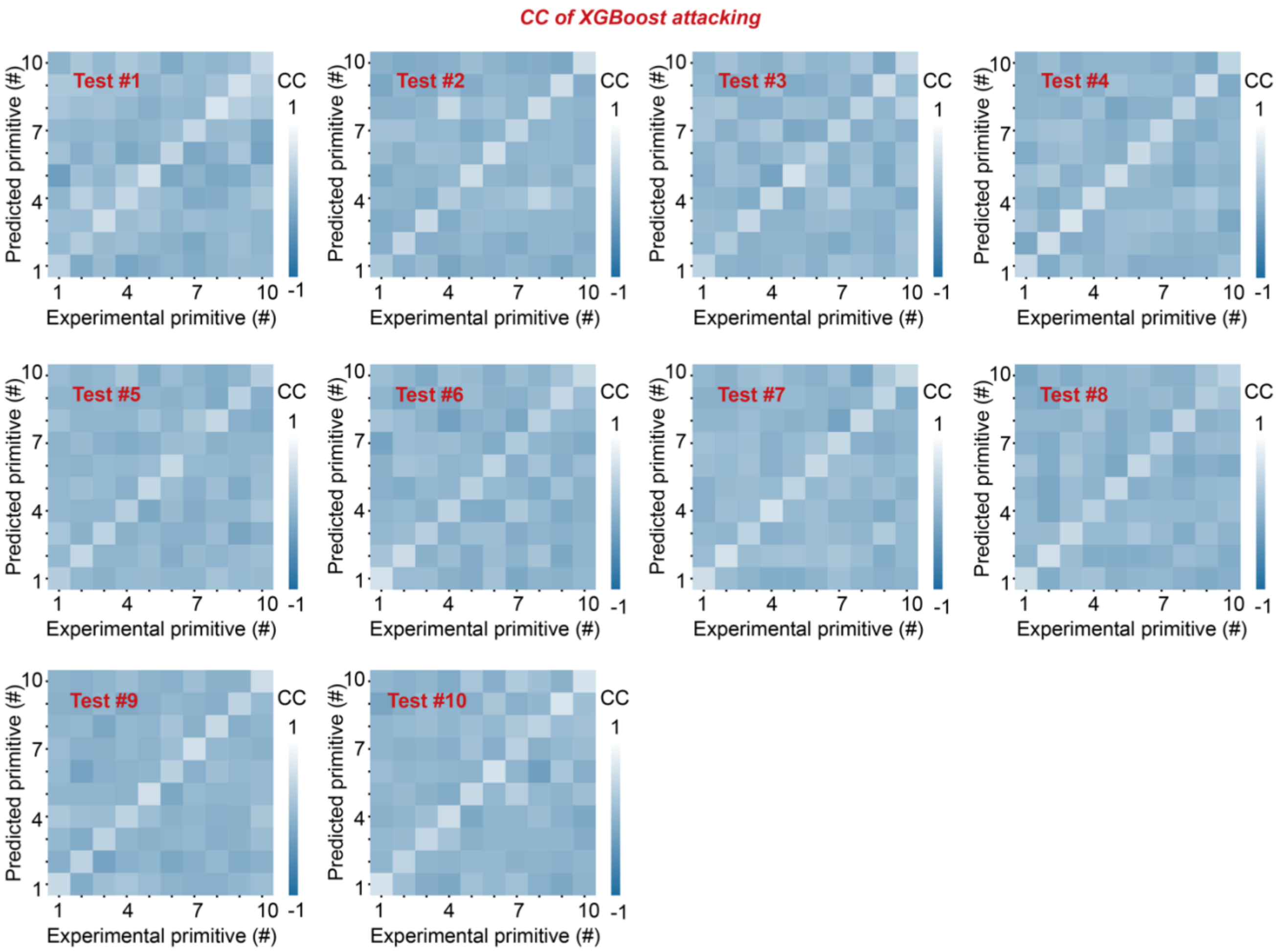

Supplementary Fig. 16. CC of XGBoost attacking in 10 tests. CC matrix of 10 experimental primitives and 10 predicted primitives as randomly and respectively selected from the 10 tests for comparison and evaluation, proving that the experimental and predicated primitives are highly irrelevant to each other, i.e. proving the resilience of the PUFs against XGBoost attacking.

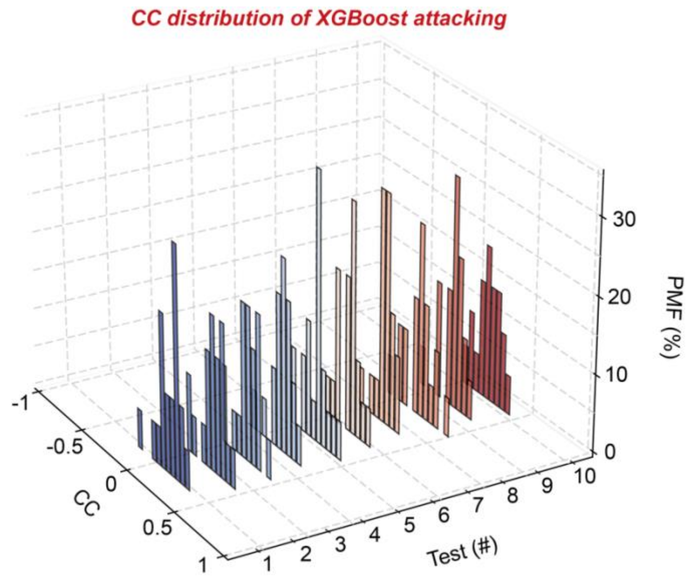

Supplementary Fig. 17. Distribution of CC of XGBoost attacking in 10 tests. Distribution of CC as analysed from the 10 experimental primitives and 10 predicted primitives as randomly and respectively selected from the 10 tests for the investigation. CC is all approaching 0.18, proving that the experimental and predicated primitives are highly irrelevant to each other, i.e. proving the resilience of the PUFs against XGBoost attacking.

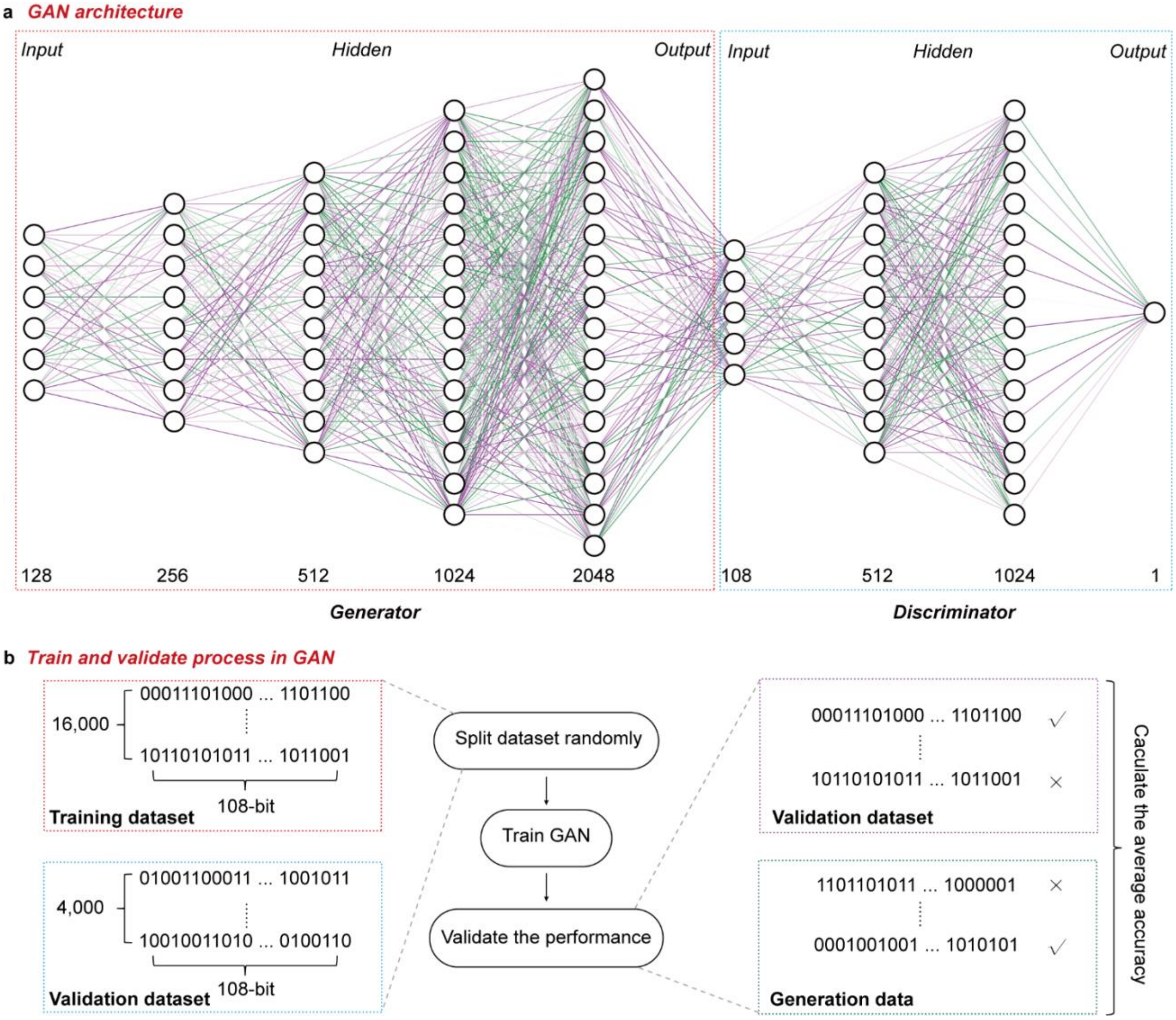

Supplementary Fig. 18. GAN network topology and the attacking diagram. (a) Architecture of the GAN network topology. (b) Schematic attacking diagram showing the training and validation of GAN.

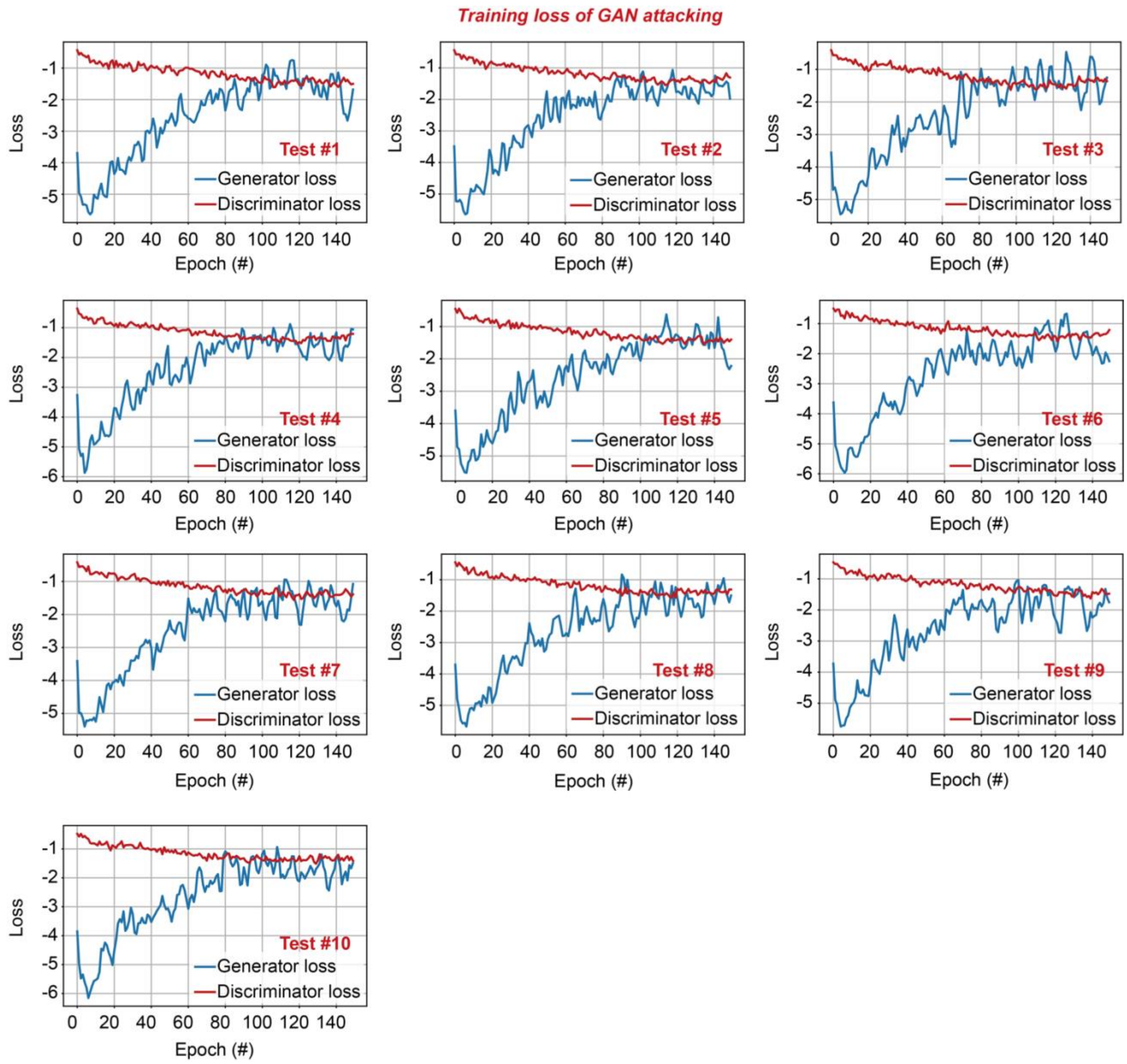

Supplementary Fig. 19. Training loss of GAN attacking in 10 tests. The training loss from the *generator* and *discriminator* in GAN attacking in the 10 tests. Note that the *Wasserstein* GAN is adapted here. It uses a batch network that produces unbounded real-valued outputs rather than probabilistic outputs. As a result, both the *generator* and *discriminator*, i.e. batchers in a *Wasserstein* GAN, can end up with negative losses. The *Wasserstein* objective calculates the difference between the average scores of the critics on the experimental and predicted primitives. It is not concerned with whether the value of non-negative losses decreases over time, but rather checks whether the "*Wasserstein* distance", i.e. the difference in the critic scores, decreases as training proceeds. This approach yields more stable training dynamics and better gradients, making *Wasserstein* GAN an attractive option for generating primitives.

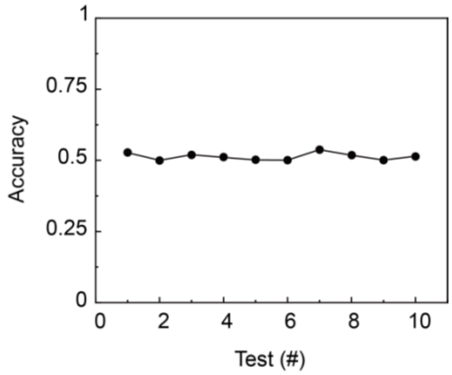

Supplementary Fig. 20. Prediction accuracy of GAN attacking over 10 tests. The averaged prediction accuracy for the 10 tests is 51.31%, proving the resilience of the PUFs against GAN attacking.

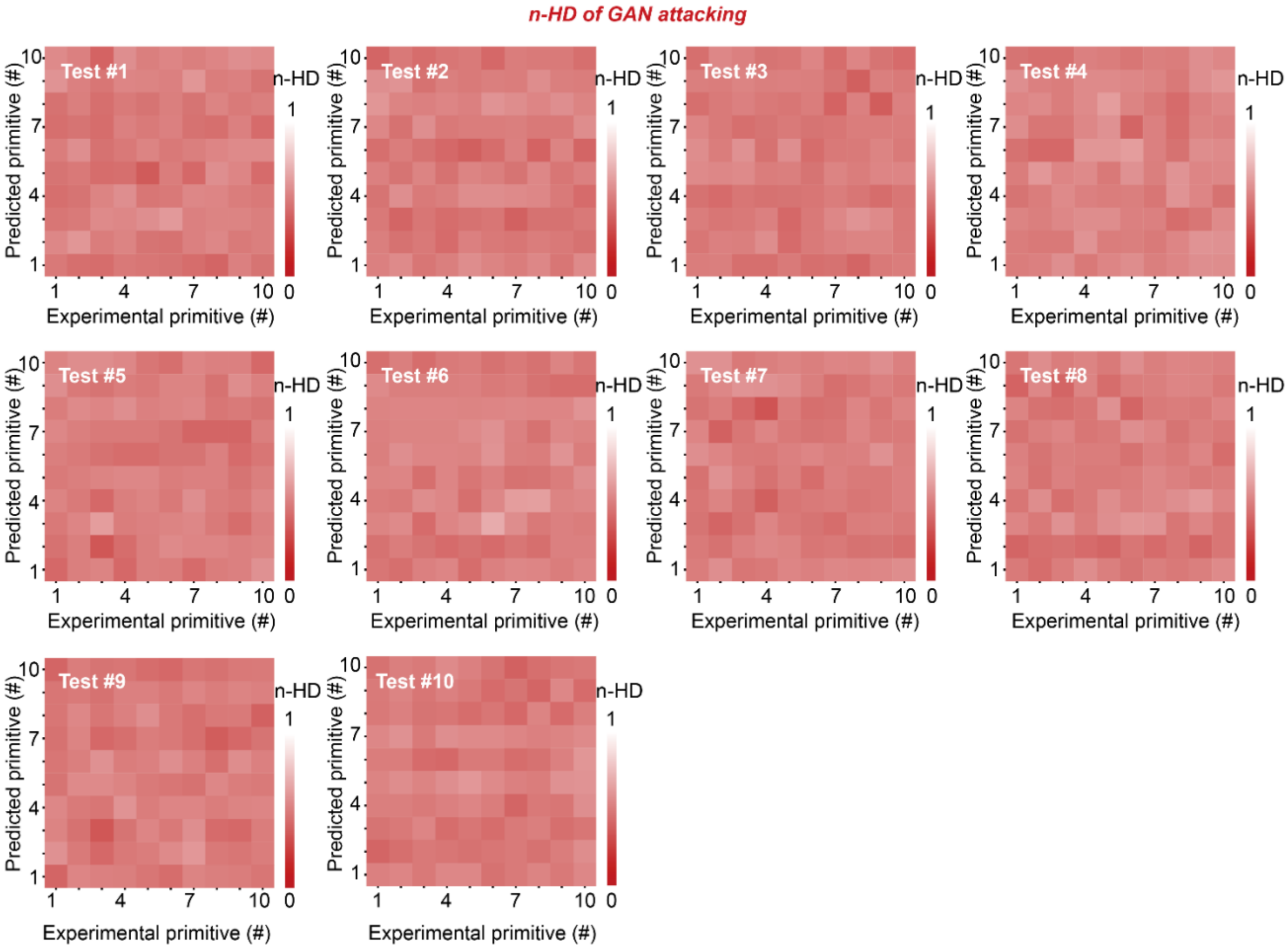

Supplementary Fig. 21. n-HD of GAN attacking in 10 tests. n-HD matrix of 10 experimental primitives and 10 predicted primitives as randomly and respectively selected from the 10 tests for comparison and evaluation, proving that the experimental and predicated primitives are highly unique to each other, i.e. proving the resilience of the PUFs against GAN attacking.

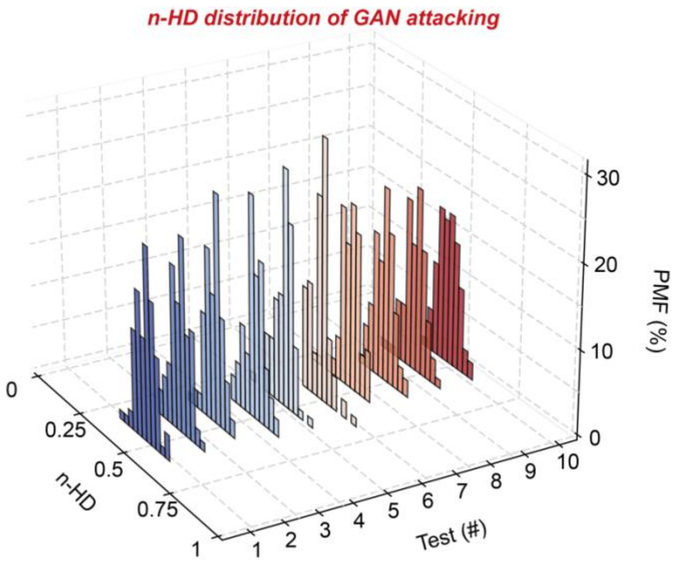

Supplementary Fig. 22. Distribution of n-HD of GAN attacking in 10 tests. Distribution of n-HD as analysed from the 10 experimental primitives and 10 predicted primitives as randomly and respectively selected from the 10 tests for evaluation. n-HD is all approaching 0.47, proving that the experimental and predicated primitives are highly unique to each other, i.e. proving the resilience of the PUFs against GAN attacking.

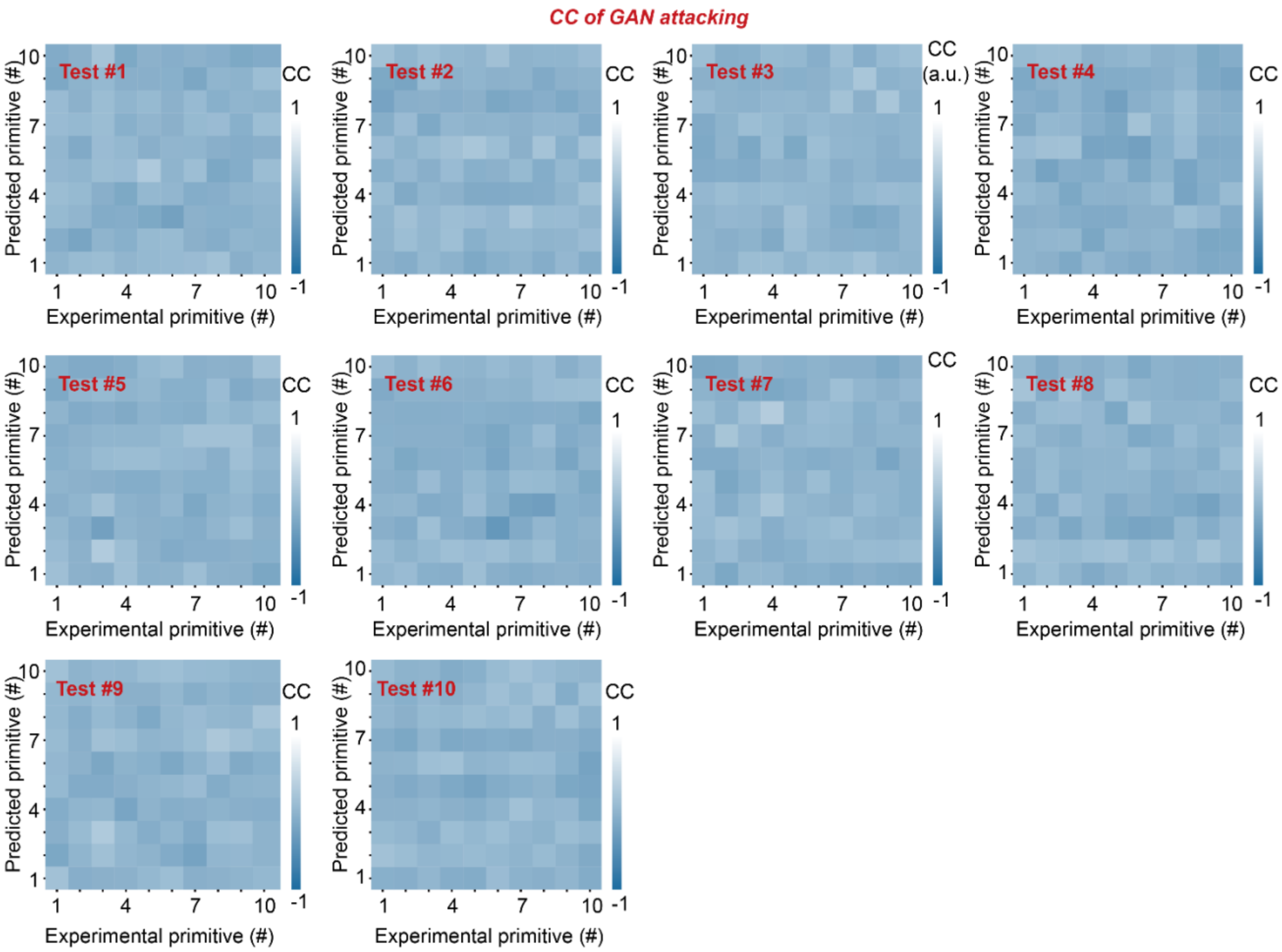

Supplementary Fig. 23. CC of GAN attacking in 10 tests. CC matrix of 10 experimental primitives and 10 predicted primitives as randomly and respectively selected from the 10 tests for comparison and evaluation, proving that the experimental and predicated primitives are highly irrelevant to each other, i.e. proving the resilience of the PUFs against GAN attacking.

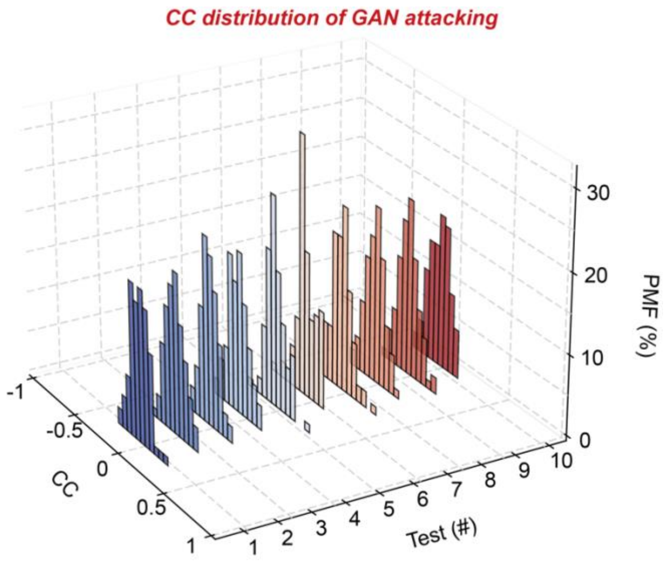

Supplementary Fig. 24. Distribution of CC of GAN attacking in 10 tests. Distribution of CC as analysed from the 10 experimental primitives and 10 predicted primitives as randomly and respectively selected from the 10 tests for evaluation. CC is all approaching 0.05, proving that the experimental and predicated primitives are highly irrelevant to each other, i.e. proving the resilience of the PUFs against GAN attacking.

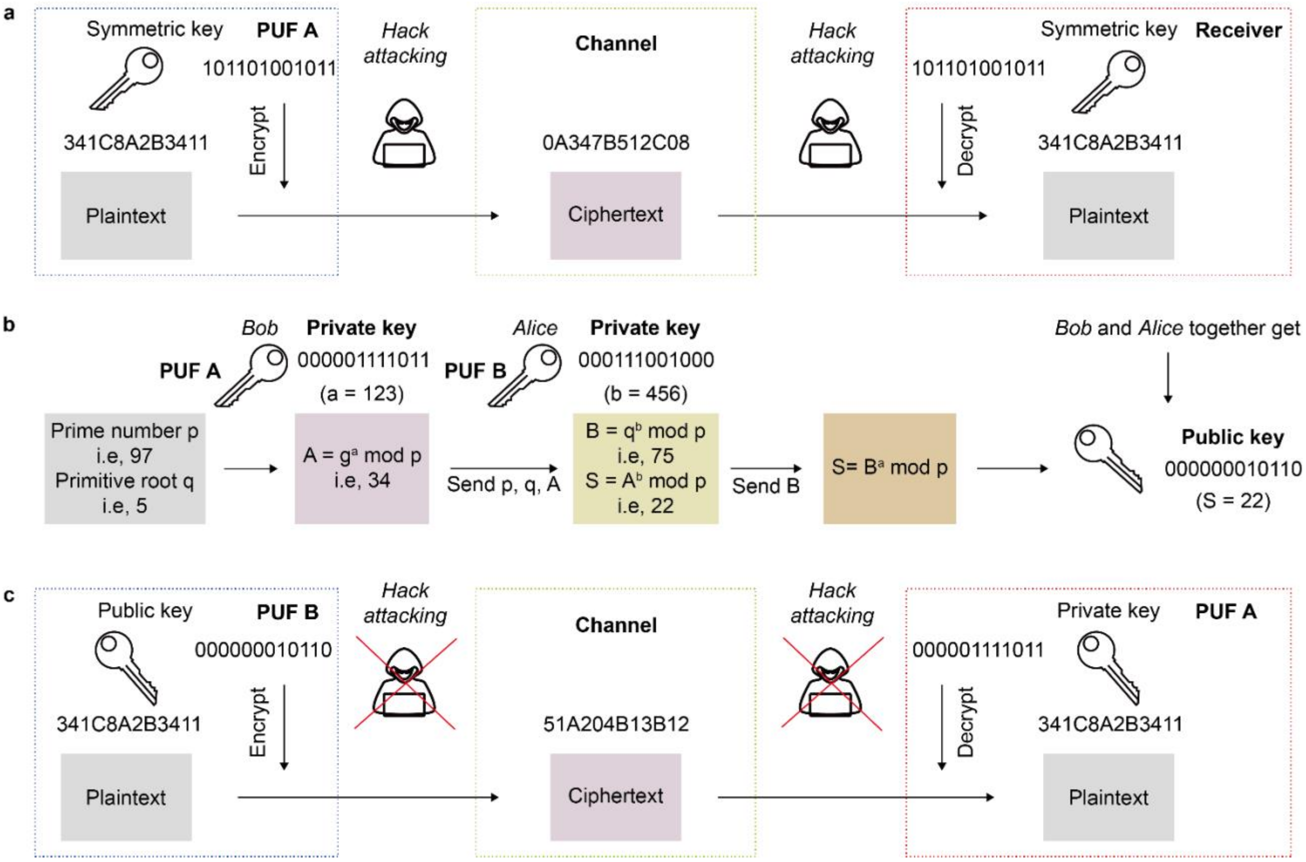

Supplementary Fig. 25. Secure communications using PUF-based key exchange protocols. The protocols are based on (a) symmetric key, (b) private-to-public key, and (c) public-to-private key exchange approaches. The protocols are based on the prime factorisation theorem and use Alice and Bob communications as the example.[3]

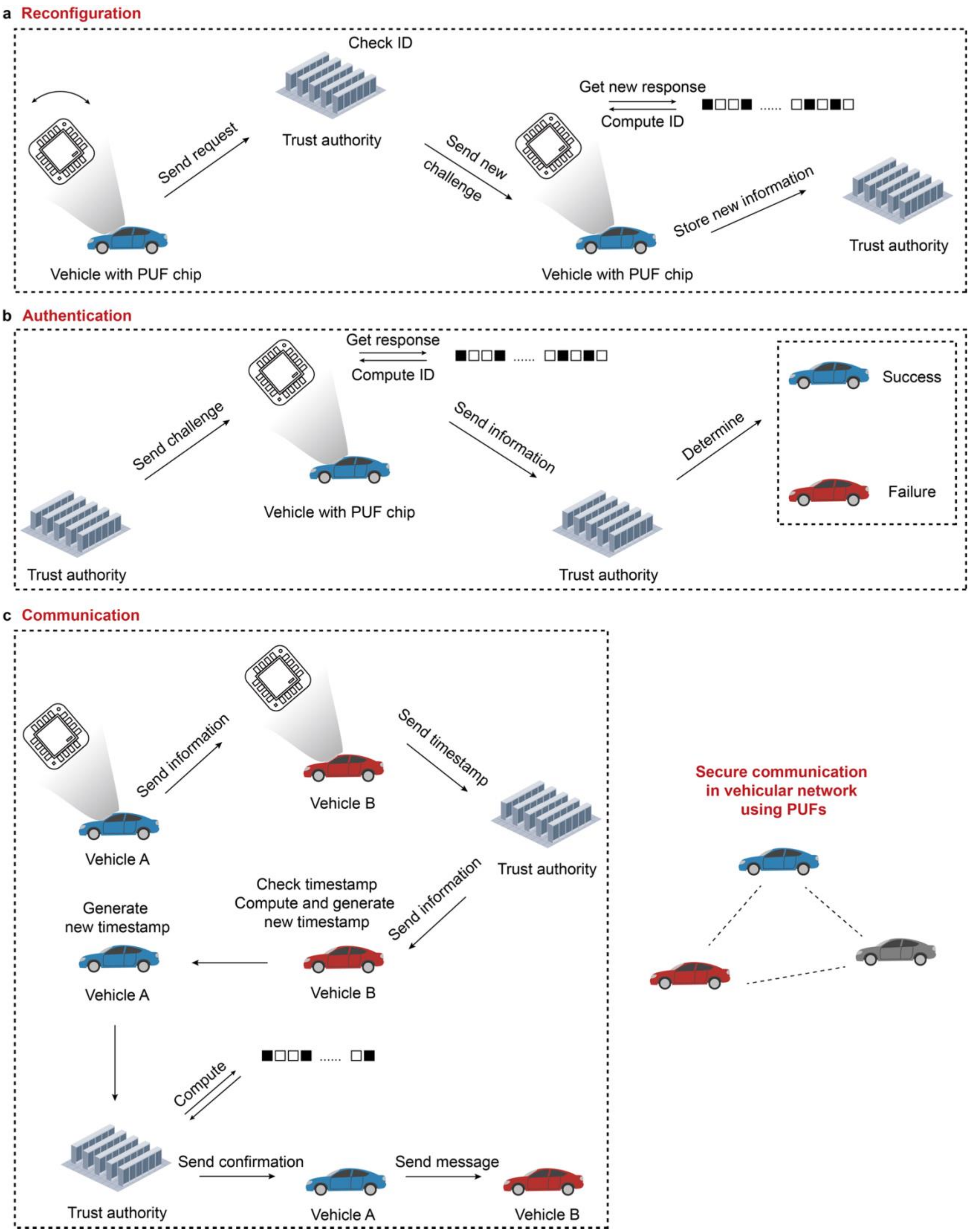

Supplementary Fig. 26. Flowchart of how PUFs play an important role in the vehicular network systems. The secured vehicular communication includes (a) reconfiguration, (b) authentication, and (c) communication processes, where the PUF chips are embedded to enable the key exchange protocol as described in Supplementary Note 1.

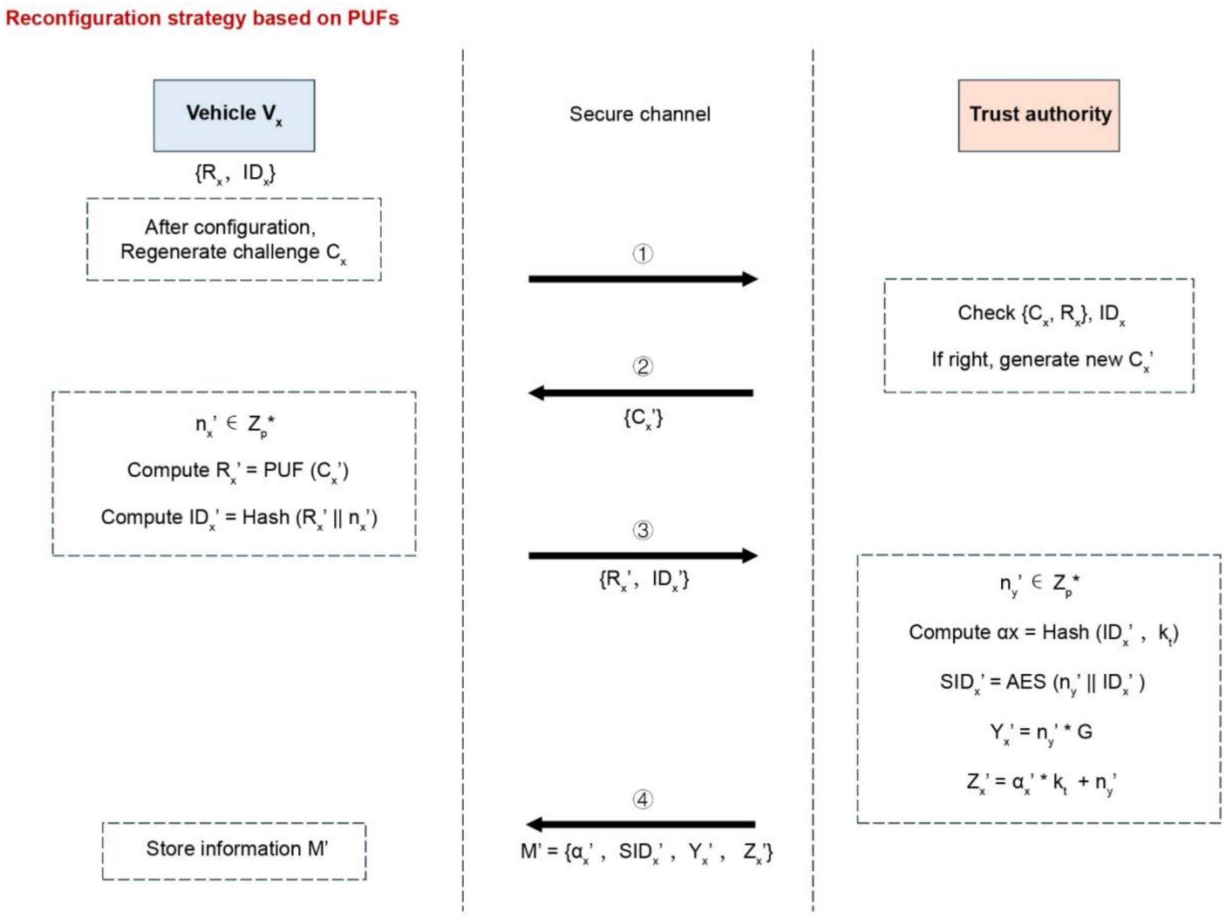

Supplementary Fig. 27. Schematic agreement diagram showing the reconfiguration between the self-driving vehicles using the PUF-based key exchange protocol. After configuration, the vehicle V_x changes its identity. V_x then regenerates the challenge to the trust authority. Trust authority checks the initial primitive, and if it agrees with the initial primitive, the trust authority sends the new challenge to V_x. V_x then regenerates a primitive to send to the trust authority to complete the reconfiguration process. PUFs are used to generate unique primitives as challenged for the reconfiguration.[2]

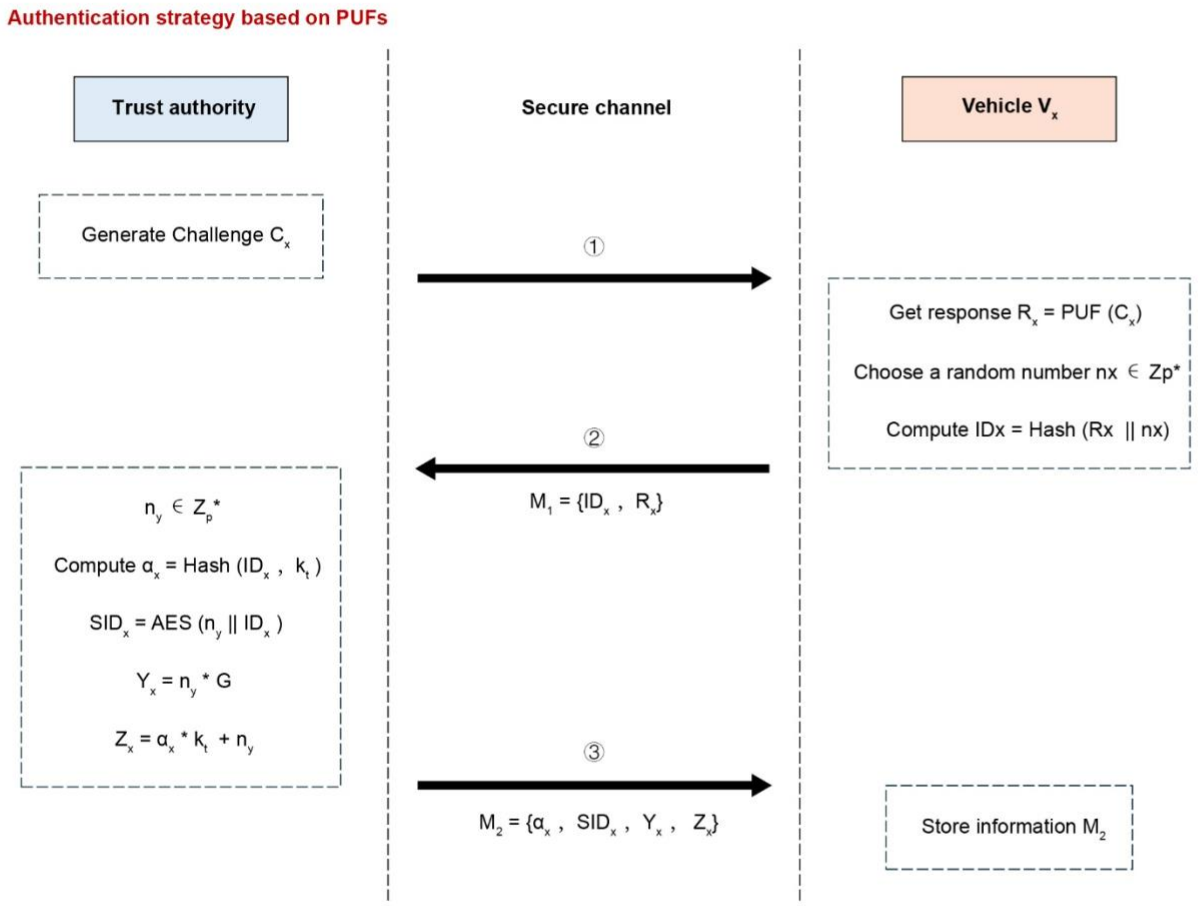

Supplementary Fig. 28. Schematic agreement diagram showing authentication of the self-driving vehicles using the PUF-based key exchange protocol. Trust authority stores the PUF primitives along with the reconfiguration and challenge instructions. When the authentication starts, the trust authority sends the reconfiguration and challenge instruction to the vehicle V_x for PUF primitive generation, and then V_x sends the primitive back to the trust authority for authentication of its identity. PUFs are used to generate unique primitives as challenged for the authentication.[2]

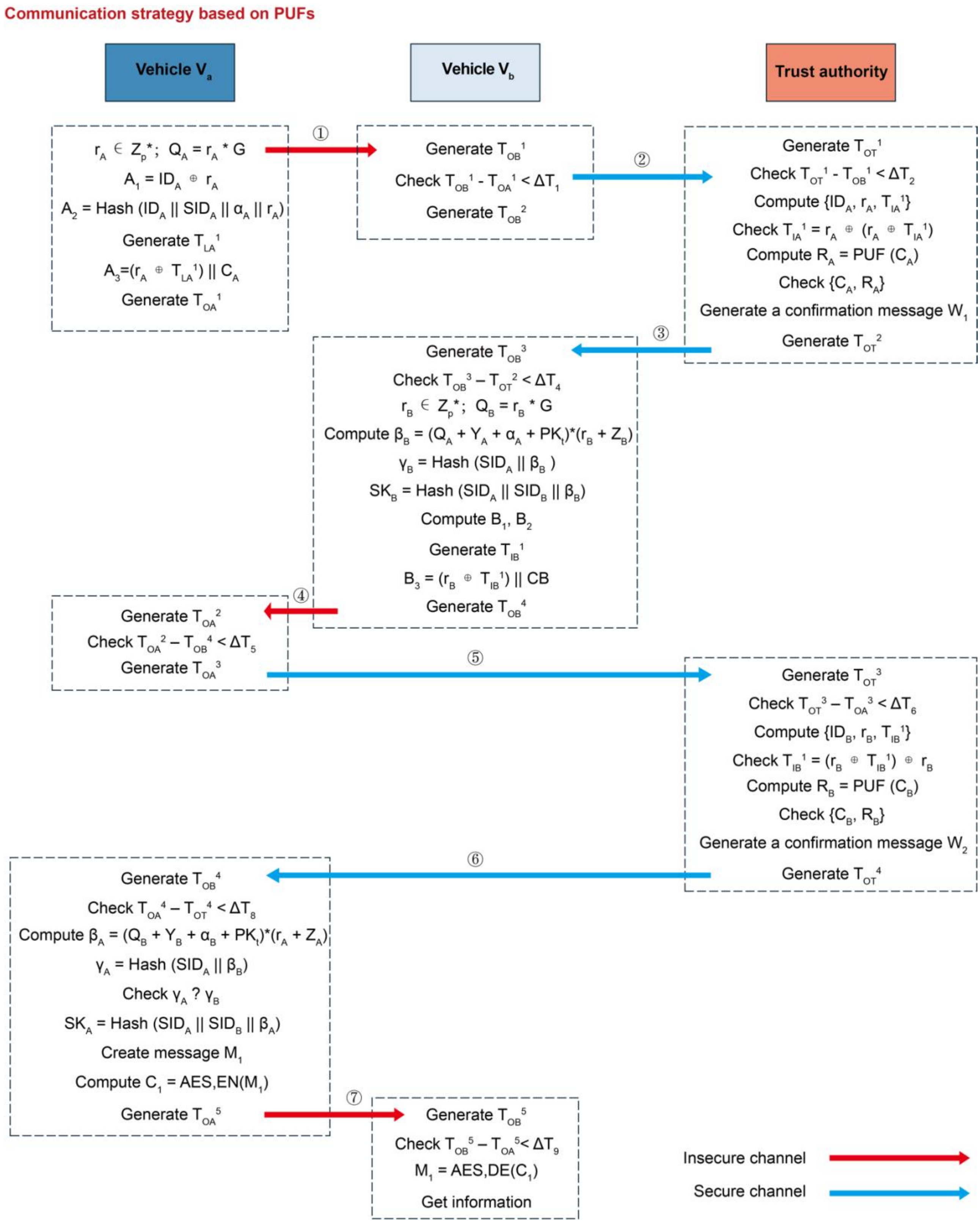

Supplementary Fig. 29. Schematic agreement diagram showing communication between the self-driving vehicles using the PUF-based key exchange protocol. Vehicle V_a and V_b, and the trust authority communicate with each other through secure and insecure channels. PUFs are used to generate unique primitives as challenged for securing the data transmission and exchange. With the PUF primitives and universal security algorithms, as well as the time stamp ensuring the freshness, the security of data transmission and exchange is guaranteed.[2]

Supplementary Table 1. Comparison between our PUF and other reported PUFs.

Materials and /or devices	Reconfigurability	Mechanism	Performance matrix	Temperature window (K)	Power consumption	Cracking time (years)	Attacking resilience	Ref.
Silicon and erbium-doped Si quantum dots	No	Structural disorder of Si metasurface and random spatial distribution of Er-Si QDs	n-HD: 0.5004 similarity: < 15%	300	/	/	/	4
Silicon	No	Standard arbiter PUF, Ring oscillator PUF, XOR arbiter PUF, etc.	/	/	/	/	>95%	5
PDMS on Si/SiO_2	No	Randomly distributed craters by infrared laser	Uniformity: 0.5 n-HD: 0.48541 - 0.49734	/	40 mW laser	/	/	6
Block copolymer	No	Non-deterministic molecular self-assembly	Uniqueness: ~0.419 - 0.506	300	/	/	/	7
Fibres in silk	No	Chaotic diffractions of randomly distributed fibres in silk	n-HD: 0.4972 - 0.4990	303 - 309	/	5×10^{41}	/	8
Quantum dots in the inkjet droplet	No	The stochastic pinning points at the three-phase	/	300	/	/	/	9

		contact line of ink						
Au network	No	Fractal-guided film annealing	n-HD: ~0.43	230 - 1045	/	/	/	10
Nanodiamonds	No	Linear polarization modulation of randomly distributed fluorescent nanodiamonds	Similarity index: 76%	300	10 mW laser	/	/	11
Indium tin oxide transistors	No	Coffee-ring structure	n-HD: 0.496	273.15 - 353.15	0.02 μW	/	55%	12
Diamond microparticles	No	Chaotic diamond microparticles by chemical vapor deposition	n-HD: 0.5 Similarity: 49.9978%	670	/	/	/	13
Hafnium oxide memristor	No	Filamentary switching characteristic of hafnium oxide memristor	Uniqueness: ~0.495, 0.505	370	~ 10 – 1000 nW	Several hundreds	70%	14
Ag nanowire network	No	Intrinsic topology of the Ag nanowire network	n-HD: ~0.45	/	/	5×10^{41}	/	15
Memristor array PUF	No	Threshold voltage variations	Uniformity: 48.5% Uniqueness: 50.1%	300	425 nW	/	/	16

Memristor-based PUF and TRNG chip	No	the forming speed and voltage variation	Uniformity: 50.09% Uniqueness: 50.06%	233.15 - 398.15	/	/	/	17
Memristor PUF	No	Cylec-to-cycle variation	n-HD: 0.496	/	/	/	49%-52%	18
Propyl pyridinium lead iodide	Yes	Intrinsically ionic and electronic coupled sources of entropy	n-HD: 0.481 - 0.4827	300 - 358	/	9.8×10^{11}	~50.5% - 52%	19
Graphene transistors	Yes	Disorders in the carrier transport of graphene field-transistors	n-HD: 0.47 CC: ~0.2	100 - 380	~ 10 mJ – 100 mJ	/	52.5%	20
**This work**	**Yes**	Charge trapping dynamics	**n-HD: ~0.5 CC: ~0**	**100-400**	**~ 34.992 fJ**	**~10^{16}**	**51.31%**	/

Note that the power consumption of this work in this table considers the consumption from the PUF only.

Supplementary Table 2. Obtained values of our reconfigurable PUFs.

	Entropy	n-HD	CC
**Fig. 3a**	0.98 ± 0.03	0.49 ± 0.05	0.02 ± 0.10
**Fig. 3b**	0.98 ± 0.03	0.48 ± 0.06	0.03 ± 0.11
**Fig. 3c**	/	0.48 ± 0.09	/
**Fig. 3d**	Index #1:<br>0.98 ± 0.03<br>Index#2:<br>0.95 ± 0.04<br>Index#3:<br>0.93 ± 0.05<br>Index#4:<br>0.90 ± 0.07<br>Index#5:<br>0.86 ± 0.07<br>Index#6:<br>0.81 ± 0.08	Index #1:<br>0.48 ± 0.06<br>Index#2:<br>0.46 ± 0.05<br>Index#3:<br>0.42 ± 0.05<br>Index#4:<br>0.39 ± 0.04<br>Index#5:<br>0.36 ± 0.04<br>Index#6:<br>0.32 ± 0.03	Index #1:<br>0.03 ± 0.11<br>Index#2:<br>0.06 ± 0.11<br>Index#3:<br>0.11 ± 0.10<br>Index#4:<br>0.17 ± 0.09<br>Index#5:<br>0.18 ± 0.09<br>Index#6:<br>0.23 ± 0.07

Supplementary Table 3. NIST SP800-22 test results of keys based on our PUFs.

	P-value	*Proportion*	*Success*	*Post-processing*
Approximate entropy	0.299	90/90	Success	No
Block frequency	0.740	90/90	Success	No
Cumulative sums	0.987	90/90	Success	No
FFT	0.226	88/90	Success	No
Frequency	0.844	90/90	Success	No
Linear complexity	0.388	89/90	Success	No
Longest run	0.557	90/90	Success	No
Non overlapping template	0.911	89/90	Success	No
Overlapping template	0.168	88/90	Success	No
Random excursions	0.503	90/90	Success	No
Random excursions variant	0.314	90/90	Success	No
Rank	0.717	90/90	Success	No
Runs	0.168	89/90	Success	No
Serial	0.557	89/90	Success	No
Universal	0.853	90/90	Success	No

Supplementary References

1. Varga, A. OMNeT++. in *Modeling and Tools for Network Simulation*, Springer Berlin Heidelberg, Berlin, Heidelberg, 2010
2. Wang, S. et al. A Lightweight, Efficient, and Physically Secure Key Agreement Authentication Protocol for Vehicular Networks. *Electronics* **13**, 1418 (2024).
3. Stamp, M. *Information Security: Principles and Practice*. (Wiley Publishing, 2011).
4. Wang, K. *et al.* All-silicon Multidimensionally-encoded Optical Physical Unclonable Functions for Integrated Circuit Anti-counterfeiting. *Nat. Commun.* **15**, 3203 (2024).
5. Rührmair, U. *et al.* PUF Modeling Attacks on Simulated and Silicon Data. *IEEE Trans. Inf. Forensics Secur.* **8**, 1876-1891 (2013).
6. Gandla, S. *et al.* Random Laser Ablated Tags for Anticounterfeiting Purposes and Towards Physically Unclonable Functions. *Nat. Commun.* **15**, 7592 (2024).
7. Kim, J. H. *et al.* Nanoscale Physical Unclonable Function Labels Based on Block Copolymer Self-assembly. *Nat. Electron.* **5**, 433-442 (2022).
8. Kim, M. S. *et al.* Revisiting Silk: A Lens-free Optical Physical Unclonable Function. *Nat. Commun.* **13**, 247 (2022).
9. Liu, Y. *et al.* Inkjet-printed Unclonable Quantum Dot Fluorescent Anti-counterfeiting Labels with Artificial Intelligence Authentication. *Nat. Commun.* **10**, 2409 (2019).
10. Sun, N. *et al.* Random Fractal-enabled Physical Unclonable Functions with Dynamic AI Authentication. *Nat. Commun.* **14**, 2185 (2023).
11. Wang, L. *et al.* High-dimensional Anticounterfeiting Nanodiamonds Authenticated with Deep Metric Learning. *Nat. Commun.* **15**, 10602 (2024).
12. Wang, R. *et al.* Printable Epsilon-Type Structure Transistor Arrays with Highly Reliable Physical Unclonable Functions. *Adv. Mater.* **35**, 2210621 (2023).
13. Zhang, T. *et al.* Multimodal Dynamic and Unclonable Anuti-counterfeiting Using Robust Diamond Microparticles on Heterogeneous Substrate. *Nat. Commuun.* **14**, 25hs07 (2023).
14. Gao, B. *et al.* Concealable Physically Unclonable Function Chip with a Memristor Array. *Sci. Adv.* **8**, eabn7753 (2022).
15. Liu, Y. *et al.* A Novel Physical Unclonable Function Based on Silver Nanowire Networks. *Adv. Funct. Mater.* **34**, 2304758 (2024).
16. Al-Tamimi, *et al.* A. Threshold voltage based dual memristor crossbar PUF. *Aeu-int. J. Electron. C.* **175**, 155012 (2024).
17. Li, X. *et al.* A memristor-based unified PUF and TRNG chip with a concealable ability for advanced edge security. *Sci. Adv.* **11**, eadr0112 (2025).
18. Ibrahim, H. M. *et al.* Resilience evaluation of memristor based PUF against machine learning attacks. *Sci. Rep.* **14**, 23962 (2024).
19. John, R. A. *et al.* Halide Perovskite Memristors as Flexible and Reconfigurable Physical Unclonable Functions. *Nat. Commun.* **12**, 3681 (2021).
20. Dodda, A. *et al.* Graphene-based Physically Unclonable Functions that are Reconfigurable and Resilient to Machine Learning Attacks. *Nat. Electron.* **4**, 364-374 (2021).
