# Supplementary material for: Chip-scale reconfigurable carbon nanotube physical unclonable functions
Source: Nat Commun. 2025 Sep 30;16:8705. doi: 10.1038/s41467-025-63739-x (PMC12484790; doi:10.1038/s41467-025-63739-x)
Supplement: Supplementary file 2 — Description of Additional Supplementary Files [file 41467_2025_63739_MOESM2_ESM.pdf]

Description of Supplementary Information files

Supplementary Movie 1: Self-driving vehicle secure communication with carbon nanotube PUF based key exchange protocols.
